# Association of socio-demographic factors, perinatal characteristics, and hospital maternity practices with breastfeeding outcomes in the UAE

**DOI:** 10.3389/fped.2025.1465193

**Published:** 2025-08-29

**Authors:** Zainab Taha, Farid El Ktaibi, Rafiq Hijazi

**Affiliations:** ^1^ Department of Health Sciences, Zayed University, Abu Dhabi, United Arab Emirates; ^2^ Department of Mathematics, Zayed University, Abu Dhabi, United Arab Emirates

**Keywords:** exclusive breastfeeding, socio-demographic determinants, baby-friendly hospitals, infant nutrition, United Arab Emirates

## Abstract

Breastfeeding (BF) rates remain suboptimal in the United Arab Emirates (UAE), despite global and national efforts. This study examined the association of socio-demographic factors, perinatal characteristics, and hospital maternity practices with breastfeeding outcomes in the UAE. In this cross-sectional study, 1,815 participating mothers with children below the age of 2 answered structured questions related to socioeconomics, hospital practices, and BF. Multivariate analysis showed that a non-Emirati nationality and vaginal birth were significantly associated with higher initiation rates (AOR = 6.19, 95% CI 1.96–19.54 and AOR = 2.65, 95% CI 1.35–5.21, respectively), timely initiation (AOR = 0.48, 95%CI 0.35–0.66, respectively), longer BF duration (AOR = 1.55, 95%CI 1.05–2.27 and AOR = 1.45, 95%CI 1.08–1.93, respectively) and longer exclusive BF duration (AOR = 1.50, 95%CI 1.06–2.11 and AOR = 1.35, 95%CI 1.03–1.78, respectively). Additionally, parity, hospital practices, maternal education, and employment were significantly associated with certain BF practices. The findings support continued efforts to implement WHO's baby-friendly initiative in more hospitals in Abu Dhabi and also emphasize the importance of early and continuous antenatal education. Emirati mothers should be prioritized in these efforts as their BF practices need more attention. As maternal employment negatively influences breastfeeding duration, supportive measures such as extended maternity leave, designated expressing facilities in the workplace, and shorter working hours are crucial to promote continued breastfeeding among employed mothers.

## Introduction

Breastfeeding (BF) is the ideal method to feed infants and young children with significant benefits related to nutrition, immunology, and cognitive development (1). According to the World Health Organization (WHO), the European Society for Pediatric Gastroenterology, Hepatology and Nutrition (ESPGHAN), and the American Academy of Pediatrics (AAP), exclusive breastfeeding (EBF) is the healthiest way to feed an infant during the first four to six months of life (2–4). Despite the UAE's commitment to WHO/UNICEF infant feeding recommendations, the proportion of children meeting optimal breastfeeding targets remains suboptimal according to WHO indicators  (5). Although breastfeeding initiation rates are high, ranging from 95.6% to 98.0%, there is a steep and rapid decline in breastfeeding practices over time  (6, 7). For example, Taha et al. (6) reported that while 95.6% of mothers initiated breastfeeding, only 44.3% exclusively breastfed at six months, and just 24.6% continued breastfeeding at 12 months (6). Similarly, Radwan (7) found that although 98% of mothers initiated breastfeeding, exclusive breastfeeding dropped to 25% at six months, and continued breastfeeding declined further to 19.8% by 12 months (7). These findings highlight a critical gap between initiation and sustained breastfeeding, particularly in the second year of life. Additionally, very few children are being breastfed until 2 years of age (6).

Several factors, including socio-demographic are known to be associated with BF practices. These include economic status and income, maternal and paternal education level, maternal age, and occupation (8). Previous studies have also found that family size, parity, support from family and health providers, and access to information are factors influencing BF practices (9, 10). Furthermore, the utilization of antenatal care services, delivery mode and habits like smoking have also been linked to BF practices (11–13).

The influence of socio-economic factors on breastfeeding (BF) practices varies significantly across cultural and national contexts. In high-income countries such as the UK and Germany, higher maternal education levels have been associated with increased BF initiation and duration (14, 15). In contrast, studies from Nigeria and three Latin American countries—Brazil, Honduras, and Mexico—show that factors like delivery in health facilities, vaginal birth, multiparity, non-employment, and infant gender are more strongly associated with BF initiation and exclusive breastfeeding (EBF) duration (16, 17). In China, higher maternal education and parental occupational status have been linked to longer BF duration (18). Other determinants, including maternal age and paternal education in Norway, Finland, and Quebec, also play a significant role in BF duration (19, 20). The data related to SES predictors of breastfeeding in Abu Dhabi is limited. In the UAE, breastfeeding used to be regarded the cultural norm as recommended in the Quran (21). However, the traditional nomadic lifestyle has changed, and the UAE today is an economically developed society housing many nationalities and cultures, with a population that consists of 81% expats (22). Breastfeeding rates in the UAE are known to be suboptimal (23).

Previous studies using this dataset have explored various outcomes and reported significant findings (24–27). However, the current study investigates new associations that were not addressed in those earlier analyses, offering a novel perspective.

### Aim

The aim of this study was to determine the association of socio-demographic factors, prenatal characteristics, and hospital maternity practices with breastfeeding outcomes in Abu Dhabi, UAE.

## Materials and methods

### Participants and data collection

**Figure 1:**
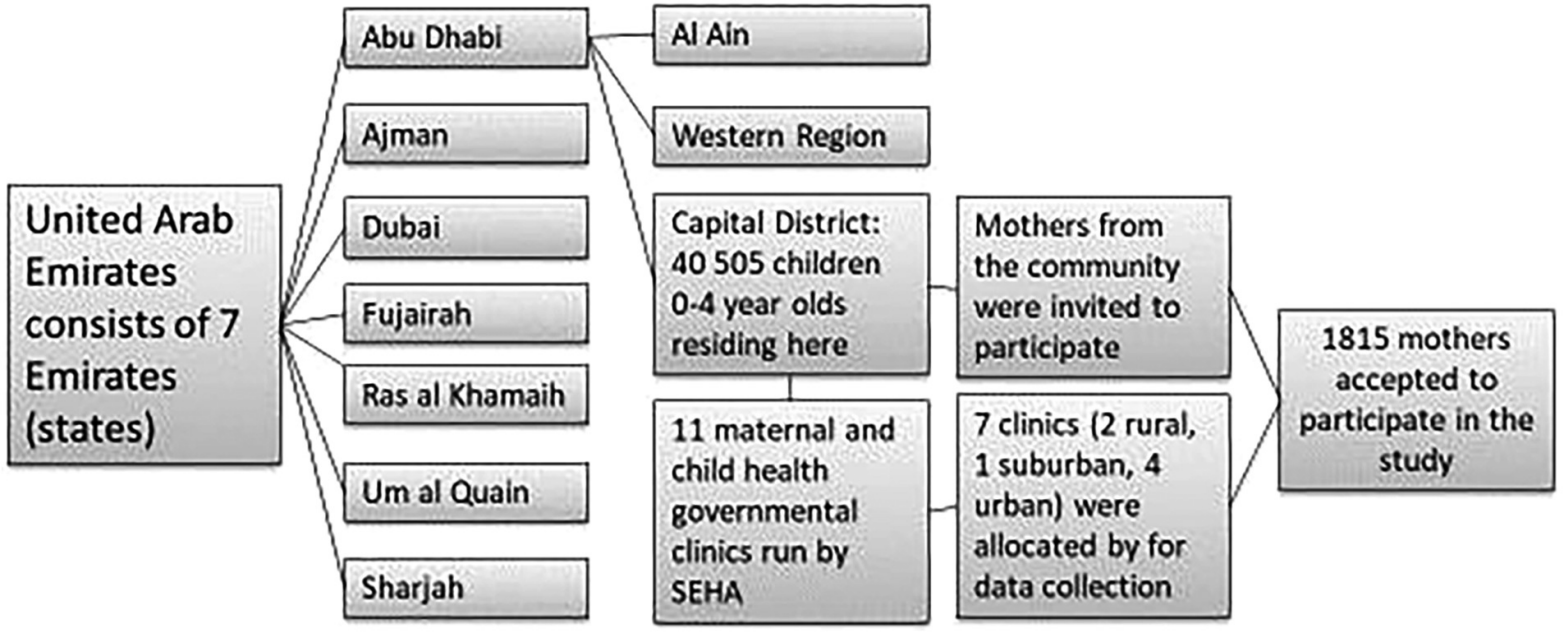
Sampling process.

The sampling process for this cross-sectional study, which was conducted in the capital district of Abu Dhabi, UAE, included both Emirati and non-Emirati families (Figure 1). More details can be found in a previous paper (6). Health insurance is mandatory in the UAE, thus both groups had access to health care (22).

Trained bilingual (Arabic and English) research assistants (RAs) approached mothers both at the health care centers and in the community at different times and days from March- September 2017. All eligible mothers who met the inclusion criteria of having at least one healthy child under the age of 2 years were invited to participate in the study. Oral and written information about the study was given to the participants by the RAs. Once the mothers had consented to participate in the study, they were interviewed by RAs using a structured questionnaire.

Study instrument (The full questionnaire provided as Supplementary Material is in English for the reader's reference).

The questionnaire, available in Arabic and English, consisted of 57 main questions with several sub-items. To ensure the validity of the questionnaire, a cross-translation technique was used where the original English questionnaire was translated into Arabic by a native Arabic speaker, followed by a retranslation back into English by a second native Arabic speaker who was blind to the original document (28, 29).

The questionnaire collected data on socio-demographics and infant-related information. It also included questions related to infant's feeding practices such as the initiation of breastfeeding, duration of exclusive and any breastfeeding, formula feeding, and the age at which complementary feeding was introduced. For the purpose of this study, the analysis focused on selected variavles with established reference to infant feeding outcomes. These included seven socio-demographic variables (maternal and paternal education, occupation, maternal age, nationality, family financial status, parity), two perinatal variables (delivery mode and gestational age), three hospital maternity practices (baby-friendly hospitals, type of facility where the birth occurred, e.g., government hospital, private hospital, and rooming-in), and three breastfeeding outcomes (initiation of breastfeeding, exclusive breastfeeding, and breastfeeding duration).

### Data analysis/statistical analysis

Following the recommendation by WHO, breastfeeding initiation and the starting time of BF within one hour after birth were used as the first two dependent variables. Exclusive breastfeeding is recommended for 6 months according to the WHO. However, based on previously reported findings in this study, very few mothers had EBF for 6 months (6). Therefore, it was decided that EBF for 3 months would be used as the third dependent variable. The recommended breastfeeding duration is 2 years, but based on previously reported findings, very few mothers breastfed beyond 12 months. Therefore, it was decided that breastfeeding duration for 6 and 9 months, respectively, would be used as the fourth dependent variable. The variables for breastfeeding duration at 6 months and at 9 months were treated as two separate dependent variables and not combined into a single “6–9 months” category. This approach was chosen to better capture the gradual decline in breastfeeding rates at different time points within the first year.

Data were analyzed using STATA version 12 (StataCorp, College Station, TX). Simple and multivariate binary logistic regression models were constructed to examine the association of the socio-demographics, hospital practices, and health among mothers with the breastfeeding practices (BF initiation, BF starting time, EBF duration, Any BF duration). Additionally, simple and multivariate ordinal logistic regression models were considered to identify the association between the aforementioned factors and BF duration.

To control for potential confounding, multivariate logistic and ordinal regression models included variables that were identified in the literature as important confounders of breastfeeding outcomes. These variables comprised maternal and paternal education, maternal employment status, maternal nationality, family financial wellbeing, parity, gestational age, mode of delivery, hospital type (baby-friendly vs. non-baby-friendly), rooming-in, and timing of breastfeeding initiation (within one hour of birth). Adjusting for these variables allowed us to estimate the independent associations between the predictors and breastfeeding outcomes.

## Results

One thousand eight hundred and fifteen mothers participated in the study. The majority of the mothers were married and had a university education. More than 60% of the participants reported their family's financial well-being as very good to excellent. One-third of the participants were of Emirati nationality, whereas the other mothers were of non-Emirati Arab or non-Emirati-non Arab origin. Most of the children were born full-term via vaginal birth. About half of the children were born in baby-friendly hospitals, and two-thirds of the mothers initiated BF within one hour (Table 1).

**Table 1:** Sample characteristics of mothers and children. *N* = 1,815.

Characteristic	Number	%
Mother's Age^a^		
17–24	217	12
25–34	1,135	62.8
35–51	455	25.2
Marital Status^a^		
Not Married	26	1.4
Married	1,784	98.6
Mother's Nationality		
Emirati	595	32.8
Non-Emirati-Arab	608	33.5
Non-Emirati-Non-Arab	612	33.7
Mother's Employment Status^a^		
Not Employed	1,164	64.2
Employed	649	35.8
Mother's Education^a^		
Below high school	80	4.4
High School	338	18.8
College/University	1,380	76.8
Father's Education^a^		
Below high school	39	2.2
High School	206	11.4
College/University	1,566	86.5
Family Financial Well-Being^a^		
Very Poor/Poor/Fair	118	6.5
Good	486	26.8
Very Good. Excellent	1,209	66.7
Parity		
1st child	649	35.8
2nd child	546	30.1
3rd child	305	16.8
4th child or more	315	17.3
Gestational Age^a^		
At most 37 weeks	228	12.6
At least 38 weeks	1,585	87.4
Delivery Type^a^		
Cesarean section	563	31
Vaginal	1,251	69
Hospital		
Non Baby friendly hospital	1,030	56.9
Baby friendly hospital	780	43.1
Rooming in^a^		
No	89	5
Yes	1,687	95
Starting Time of B^F^^a^		
More than one hour	652	37.5
Within one hour	1,086	62.5

^a^ There is some missing data in these variables.

Table 2 presents the associations between initiation of breastfeeding and socio-demographics, hospital practices, and health variables among mothers from Abu Dhabi, UAE. Mothers' age, marital status, fathers’ education, family financial wellbeing, gestational age, and hospital type were not significantly associated with whether mothers initiated breastfeeding. In the unadjusted univariate analysis, mothers’ nationality, maternal employment, delivery type, and rooming-in were associated with breastfeeding initiation (Table 2). Some of these significances disappeared when adjusting for confounding variables. In the multivariate logistic regression analysis, after adjusting for confounding factors, it was found that the non-Emirati-non-Arab mothers were significantly more likely to initiate breastfeeding compared to Emirati mothers As for the parity, mothers who had their fourth child were significantly more likely to initiate breastfeeding compared to mothers who had their first child Mothers who had a vaginal birth were significantly more likely to initiate breastfeeding compared to those having a cesarean section Mothers in hospitals with rooming-in were significantly more likely to initiate breastfeeding compared to those who delivered in hospitals without rooming-in On the other hand, mothers with high school education were less likely to initiate breastfeeding compared to mothers with a higher education level (Table 2).

**Table 2:** Significant associations between the initiation of breastfeeding and socio- demographics variables, hospital practices and health variables among mothers from Abu Dhabi, UAE.

BF Initiation	Unadjusted				Adjusted			
	OR	*p*-value	95% CI		OR	*p*-value	95% CI	
Mother's Nationality								
Emiratis	1							
Non-Emirati-Arab	1.58	0.140	0.86	2.90	1.91	0.115	0.85	4.27
Non-Emirati-Non-Arab	5.87	**0.000**	2.24	15.34	6.19	**0.002**	1.96	19.54
Mother's Employment								
Not Employed	1							
Employed	0.41	**0.002**	0.24	0.73	0.52	0.065	0.26	1.04
Mother's Education								
Below high school^a^								
High School	0.70	0.271	0.37	1.33	0.40	**0.029**	0.18	0.91
College/University	1							
Parity								
1st child	1							
2nd child	0.78	0.454	0.41	1.48	0.91	0.800	0.43	1.93
3rd child	1.29	0.572	0.54	3.10	2.28	0.128	0.79	6.59
4th child or more	1.86	0.222	0.69	5.02	4.08	**0.043**	1.05	15.93
Delivery Type								
Caesarean section	1							
Vaginal	3.59	**0.000**	2.03	6.36	2.65	**0.005**	1.35	5.21
Rooming in								
No	1							
Yes	18.30	**0.000**	9.89	33.86	15.48	**0.000**	6.64	36.10

Bold values indicate statistically significant associations, defined as *p*-values less than 0.05.

^a^ All mothers with an education level below high school initiated breastfeeding and were therefore not included in the analysis since there was no variation.

Table 3 presents the associations between the starting time of breastfeeding after giving birth and socio-demographics, hospital practices, and health variables among mothers from Abu Dhabi, UAE. Age, marital status, and mothers’ employment status were not significantly associated with the starting time of breastfeeding. In the unadjusted univariate analysis, mothers’ nationality, mothers’ education, fathers’ education, family financial wellbeing, parity, gestational age, delivery type, hospital type, and rooming-in were significantly associated with the starting time. However, after considering confounding factors, in the AOR, non-Emirati-Arabs and non-Emirati-non-Arabs were significantly less likely to start breastfeeding within an hour than Emirati women (Table 3). Families who reported their financial wellbeing to be very good or excellent were significantly less likely to start breastfeeding within an hour than those who reported a very poor/poor or fair financial wellbeing. Mothers who had their second child were significantly more likely to start breastfeeding within an hour than those who had their first child. Similarly, those who had a vaginal delivery were significantly more likely to start breastfeeding within an hour than those who had cesarean section. Moreover, mothers who had access to rooming-in were significantly more likely to start breastfeeding within an hour than those who did not.

**Table 3:** Signficant associations between the starting time of breastfeeding after giving birth and socio-demographics, hospital practices and health variables among mothers from Abu Dhabi, UAE.

Starting time of BF after birth	Unadjusted				Adjusted			
	OR	*p*-value	95% CI		OR	*p*-value	95% CI	
Mother's Nationality								
Emiratis	1							
Non-Emirati-Arab	0.48	**0.000**	0.37	0.61	0.48	**0.000**	0.35	0.66
Non-Emirati-Non-Arab	0.48	**0.000**	0.37	0.62	0.46	**0.000**	0.32	0.65
Mother's Education								
Below high school	1							
High School	0.46	**0.009**	0.26	0.83	0.54	0.070	0.28	1.05
College/University	0.46	**0.005**	0.26	0.79	0.73	0.339	0.38	1.40
Father's Education								
Below high school	1							
High School	0.48	0.124	0.19	1.22	0.46	0.142	0.16	1.30
College/University	0.33	**0.013**	0.13	0.79	0.46	0.136	0.17	1.28
Family Financial Well-Being								
Very Poor/Poor/Fair	1							
Good	1.24	0.358	0.78	1.96	1.06	0.812	0.64	1.77
Excellent/Very Good	0.55	**0.005**	0.36	0.84	0.44	**0.001**	0.27	0.71
Parity								
1st child	1							
2nd child	1.43	**0.004**	1.12	1.82	1.36	**0.026**	1.04	1.79
3rd child	1.30	0.072	0.98	1.72	1.18	0.330	0.85	1.64
≥4 child	1.87	**0.000**	1.39	2.51	1.26	0.234	0.86	1.85
Gestational Age								
≤37 weeks	1							
≥38 weeks	1.64	**0.001**	1.23	2.19	1.25	0.196	0.89	1.77
Delivery Type								
Cesarean section	1							
Vaginal	3.14	**0.000**	2.54	3.89	2.93	**0.000**	2.32	3.70
Hospital								
Non-Baby friendly hospital	1							
Baby friendly hospital	1.23	**0.042**	1.01	1.50	0.82	0.142	0.64	1.07
Rooming in								
No	1							
Yes	3.86	**0.000**	2.28	6.52	4.49	**0.000**	2.49	8.11

Bold values indicate statistically significant associations, defined as *p*-values less than 0.05.

Breastfeeding designation (exclusive vs. any breastfeeding) was analyzed only in relation to breastfeeding duration and exclusivity (Tables 4, 5). It was not included in the analyses of breastfeeding initiation or starting time (Tables 2, 3), as designation reflects ongoing feeding patterns rather than immediate initiation outcomes.

**Table 4:** Significant associations between the breastfeeding duration and socio- demographics, hospital practices and health variables among mothers from Abu Dhabi, UAE.

BF Duration	Unadjusted				Adjusted			
	OR	*p*-value	95% CI		OR	*p*-value	95% CI	
Mother's Nationality								
Emiratis	1							
Non-Emirati-Arab	0.92	0.577	0.70	1.22	1.29	0.161	0.90	1.83
Non-Emirati-Non-Arab	1.08	0.567	0.83	1.42	1.55	**0**.**026**	1.05	2.27
Mother's Employment								
Not Employed	1							
Employed	0.72	**0**.**006**	0.57	0.91	0.65	**0**.**001**	0.50	0.85
Mother's Education								
Below high school	1							
High School	1.72	0.104	0.89	3.31	2.43	**0**.**021**	1.15	5.14
College/University	1.52	0.179	0.83	2.80	2.56	**0**.**011**	1.24	5.30
Parity								
1st child	1							
2nd child	1.42	**0**.**017**	1.06	1.88	1.41	**0**.**030**	1.03	1.93
3rd child	1.42	**0**.**046**	1.01	1.99	1.44	0.063	0.98	2.10
4th child or more	1.30	0.115	0.94	1.80	1.11	0.639	0.73	1.68
Delivery Type								
Cesarean section	1							
Vaginal	1.69	**0**.**000**	1.31	2.19	1.45	**0**.**012**	1.08	1.93
Hospital								
Non Baby friendly hospital	1							
Baby friendly hospital	1.18	0.154	0.94	1.48	1.38	**0**.**029**	1.03	1.85
Starting Time of BF								
More than one hour	1							
Within one hour	2.36	**0**.**000**	1.83	3.04	2.35	**0**.**000**	1.77	3.12

Bold values indicate statistically significant associations, defined as *p*-values less than 0.05.

**Table 5:** Significant associations between the exclusive breastfeeding duration and socio demographics, hospital practices and health variables among mothers from Abu Dhabi., UAE.

EBF Duration	Unadjusted				Adjusted			
	OR	*p*-value	95% CI		OR	*p*-value	95% CI	
Mother's Nationality								
Emiratis	1							
Non-Emirati-Arab	1.15	0.294	0.88	1.51	1.50	**0.021**	1.06	2.11
Non-Emirati-Non-Arab	1.59	**0.001**	1.22	2.08	2.18	**0.000**	1.49	3.19
Mother's Employment								
Not Employed	1							
Employed	0.75	**0.014**	0.60	0.94	0.73	**0.017**	0.57	0.95
Family Financial Well-Being								
Very Poor/Poor/Fair	1							
Good	0.75	0.209	0.47	1.18	0.72	0.192	0.43	1.18
Excellent/Very Good	0.62	**0.029**	0.40	0.95	0.66	0.086	0.41	1.06
Gestational Age								
≤37 weeks	1							
≥38 weeks	1.53	**0.011**	1.10	2.13	1.26	0.226	0.87	1.83
Delivery Type								
Cesarean section	1							
Vaginal	1.50	**0.001**	1.18	1.91	1.35	**0.032**	1.03	1.78
Rooming in								
No	1							
Yes	2.07	**0.016**	1.14	3.73	1.24	0.519	0.64	2.40
Starting Time of BF								
More than one hour	1							
Within one hour	2.57	**0.000**	2.03	3.26	2.50	**0.000**	1.91	3.26

Bold values indicate statistically significant associations, defined as *p*-values less than 0.05.

Table 4 presents the associations between the breastfeeding duration and socio- demographic factors, hospital practices and health variables among mothers from Abu Dhabi. None of the following factors showed a significant association with breastfeeding duration: mothers’ age, marital status, fathers’ education level, family financial wellbeing, gestational age or rooming-in.

In the unadjusted univariate analysis, mothers’ employment, parity, delivery type and starting time of delivery were significantly associated with breastfeeding duration (Table 4). In the multivariate analysis, after adjusting for confounding factors, it was found that non-Emirati-non-Arabs were significantly more likely to have a longer breastfeeding duration than Emirati mothers’ whereas employed mothers’ were significantly less likely to have a long breastfeeding duration than mothers’ who were housewives or students.

Mothers’ with a high school or university education were more likely to continue breastfeeding for a longer duration than those with an education below high school. Likewise, mothers who had given birth to their second child were significantly more likely to have longer breastfeeding duration than those who had given birth to their first child Women who had a vaginal delivery vs. cesarean section and those giving birth at baby-friendly hospitals rather than traditional hospitals were significantly more likely to have a longer breastfeeding duration Finally, mothers who started breastfeeding within an hour after giving birth were significantly more likely to have longer breastfeeding duration than those who started breastfeeding later (Table 4).

Table 5 presents the associations between the exclusive breastfeeding duration and socio-demographics, hospital practices and health variables among mothers from Abu Dhabi, UAE. There was no significant association between exclusive breastfeeding duration and mothers’ age, marital status, parental education, parity, or hospital type. In the unadjusted univariate analysis, significant associations with exclusive breastfeeding duration were observed for mothers’ nationality and employment, family financial wellbeing, gestational age, delivery type, rooming-in, and timing of breastfeeding initiation (Table 5). In the multivariate analysis, after adjusting for confounding factors, it was found that both non-Emirati-Arabs and non-Emirati-non-Arabs were significantly more likely to have longer exclusive breastfeeding duration than Emirati mothers Women who had a vaginal delivery were significantly more likely to exclusively breastfeed for a longer duration compared to those who had a cesarean section, as were those who initiated breastfeeding within an hour after birth compared to those who started later. In contrast, employed mothers were significantly less likely to breastfeed exclusively for a longer duration compared to those who were not employed.

## Discussion

The current study demonstrated significant associations between breastfeeding practices and socio-demographic factors in Abu Dhabi. A non-Emirati nationality and vaginal birth were positively associated with initiation rate, timely initiation, EBF, and BF duration. Additionally, parity, hospital practices, maternal education, and employment were significantly associated with certain BF practices, whereas maternal age, paternal education, and employment, or family financial wellbeing showed no significant or limited association with BF practices.

In this study hospital practices played an important role in the breastfeeding outcomes. Vaginal birth was positively associated with breastfeeding initiation, timely initiation, EBF, and the BF duration. Similar results have been found in neighboring countries with similar cultures (30–33). The increased rates of C-sections in the UAE over the past 20 years are therefore concerning, not only for the mother, but also for neonatal health, as they have been associated with suboptimal breastfeeding practices (34).

In this study, rooming-in had a significant positive association with the initiation rate and the timely initiation of breastfeeding, which is similar to other regional studies (7, 31, 35). Subsequently, a timely initiation was associated with significantly longer BF and EBF durations. A systematic review found positive associations between birth giving in baby-friendly hospitals and breastfeeding duration, which supports our findings (36). These results suggest that continued efforts by the health authorities in increasing the numbers of accredited baby-friendly hospitals in Abu Dhabi, including deliberate efforts in implementing step 10-the community support- may help promote longer breastfeeding duration.This variable previously has been rated “poorin the UAE according to the WHO’s infant feeding indicators” (6, 36).

In other regions, socio-economic factors have been associated with BF outcomes. In this study, non-Emirati mothers had a higher initiation rate, more timely initiation, and longer duration of exclusive and any BF compared to Emirati mothers. Previous research has also linked Emirati nationality with suboptimal health-related choices for preschool children, suggesting that Emirati families should be a prioritized group for basic health education and targeted health interventions to improve child health outcomes in the UAE (37).

Globally, the association between women's education and employment status and breastfeeding practices has shown inconsistent results (38). Although BF is the traditional infant feeding method in the Middle East, there are no conclusive results on how the education level is associated with breastfeeding practices. Findings from the current study indicate that mothers with a high school education had lower initiation rates than those with lower education levels, whereas mothers with higher education had longer BF duration compared to those with lower education. This is consistent with a study in Saudi Arabia but contrasts with findings from a study in Kuwait, which reported a significant negative association between breastfeeding duration and maternal education (32, 39).

As demostreted in this and other similar studies in the Middle East, BF initiation rates (40, 41) are often high. However, one of the main challenges in meeting the UNICEF/WHO infant feeding goals appears to lie in maintaining breastfeeding for the recommended duratio, with the exception of Iran (42). Currently, maternal employment is associated with shorter durations of EBF and any BF, which aligns with findings from other studies conducted both regionally and globally (31, 43–45). Additionally, EBF and BF duration also appear to be related to maternity leave—in terms of both financial compensation and length. For instance, in a study of 38 low and middle-income countries, a one-month increase in the legislated duration of paid maternity leave was associated with a 2.2-month increase in BF duration. Similar findings have also been reported in the United States (46, 47). Therefore, maternity leave may act as a confounding factor in the observed relationship between employment and breastfeeding outcomes, highlighting the need for further investigation. In the UAE, extending paid maternity leave, offering flexible or reduced working hours, and ensuring access to breastfeeding facilities in workplaces could be important strategies moving forward. These measures have the potential to support health-promoting breastfeeding behaviors among employed women, and over time, contribute to improved health outcomes among the young population (47).

According to Hackman et al., multiparous mothers are more likely to initiate breastfeeding and breastfeed for more extended periods than primiparous mothers (48). Consistent with this, findings from the current study showed that mothers who had given birth to their second child were significantly more likely to have a longer breastfeeding duration than those who had given birth to their first child. This highlights the need for early and continuous antenatal education, particularly targeted toward primiparous mothers.

### Strengths and weaknesses

A major strength of the study is its large sample size, which included both UAE nationals and expatriates recruited from the majority of maternal and child health centers across various geographical areas in Abu Dhabi. This enhances the generalizability of the findings and suggests that the results are likely representative of the broader population in Abu Dhabi. Another strength is the comprehensive assessment of socio-economic factors and breastfeeding practices, using WHO indicators including initiation, timely initiation, duration of EBF and any BF.

A limitation for this, and other similar studies, is related to the accuracy of long-term maternal recall of breastfeeding practices but with a majority of the children (72%) being 1 year or below, the impact should be limited (6).

While the data were collected a few years ago, they remain relevant, as no major policy changes or national breastfeeding initiatives have taken place in the UAE during this period that would significantly alter breastfeeding practices. Nonetheless, the time frame of data collection is acknowledged as a potential limitation. Although the findings likely reflect current trends, ongoing surveillance is necessary to monitor possible changes in breastfeeding behaviors over time.

## Conclusion

To conclude, both vaginal birth and rooming-in were positively associated with breastfeeding initiation and timely initiation, which in turn were linked to longer durations of both exclusive and any breastfeeding. Giving birth at baby-friendly hospitals and being a multiparous mother were also associated with longer breastfeeding durations. These findings support continued efforts to expand the WHO Baby-Friendly Hospital Initiative across hospitals in Abu Dhabi and underscore the importance of early and ongoing antenatal education, particularly for primiparous women. Compared to other nationalities, the proportionately lower rates of breastfeeding initiation and duration observed among Emirati mothers warrant further investigation to identify contributing factors and guide the development of culturally appropriate support and interventions. Additionally, the negative association between maternal employment and breastfeeding duration highlights the need to examine workplace-related supports, such as extended maternity leave, breastfeeding facilities, and flexible working hours, which have shown effectiveness in other settings and may be relevant in the UAE context.

## Data Availability

The raw data supporting the conclusions of this article will be made available by the authors, without undue reservation.
