# Supplementary material for: Association of socio-demographic factors, perinatal characteristics, and hospital maternity practices with breastfeeding outcomes in the UAE
Source: Front Pediatr. 2025 Aug 29;13:1465193. doi: 10.3389/fped.2025.1465193 (PMC12427024; doi:10.3389/fped.2025.1465193)
Supplement: Supplementary file 1 [file Datasheet1.pdf]

Title: Knowledge and Practices of Mothers on Complementary Feeding in the UAE

Principal Investigator: **Dr. Zainab Taha**, Assistant Professor (Office: +971 – 025993756)

ID:

All Information collected will generate group statistics only. Your answers are voluntary, confidential and anonymous.

**I agree to participate in this study: Your signature**		**Date of interview**	(dd/mm/yyyy) _____/_____/__________
**Name of this Child** ***(Index child for all subsequent questions asked)***		**Health Center**	
**Name of Interviewer**		**Name of Hospital where this child was delivered**	

	**Question**	**Answer**
**PART I : DEMOGRAPHIC AND HEALTH DATA FOR THE PARENTS AND THE CHILD (PLEASE CIRCLE YOUR ANSWER)**		
	**MOTHER'S INFORMATION**	
1.	How old are you?<br>Your date of birth (dd/mm/yyyy) _____/_____/__________	___ ___ years old
2.	Marital status	1) Married<br>2) Divorced<br>3) Widowed
3.	What is your nationality?	1) UAE<br>2) Other Arab Nationality<br>3) Asian<br>4) Other
4.	How would you describe yourself? **(Check all that apply)**	1) Student _______<br>2) Employed _______<br>3) Housewife _______<br>4) Other (specify) _______
5.	I you are student, which year and semester are you now enrolled	Year ___________ Semester ___________<br>University name and Major (Specify)____________________
6.	What is the highest level of education have you completed	1) Not applicable<br>2) Below High School<br>3) High School<br>4) Some College<br>5) Finished College

	Question	Answer
		6) University or Higher (Masters, Doctorate, etc)
7.	What is the educational level of the father of this child ***(Use the index child's name)?***	1) No education<br>2) Reads & writes<br>3) Primary<br>4) High school<br>5) College<br>6) University or higher
8.	How tall are you without shoes	_______ cms (e.g. 175 cms)
9.	How much do you weigh now?	_____._____ kgs (e.g. 45.7 kgs)
10.	How much did you weigh **before you became pregnant** with this baby (name)?	_____._____ kgs (e.g. 45.7 kgs)
11.	Can you tell me how much you weighed immediately after you delivered this baby (name)?	_____._____ kgs (e.g. 45.7 kgs)
12.	What is the monthly income in AED in your family?	___________________ Dirhams<br><br>Don't know ____________
13.	How many people in your house does this income support?	______________________
14.	Considering your monthly family income, how would you rate your and your family's overall financial well-being	1. Excellent<br>2. Very Good

	Question	Answer
		3. Good<br>4. Fair<br>5. Poor<br>6. Very Poor
15.	How many children of your own do you have including this child (name)?	____________ (No. of children. Use 1 if this child (name) is the only child)
16.	What is the gender (name) of this child (name)?	1) Male<br>2) Female
17.	What is the birth order of this child (name)?	1) First<br>2) Second<br>3) Third<br>4) Fourth<br>5) Fifth or more
18.	How much did this child (name) weigh at birth?	______ grams<br><br>_________I don't know
19.	What was the length of this child (name) at birth?	________ centimeters<br><br>___________I don't know
20.	What was the gestational age of this child (name) at birth:	________ weeks _________ days
21.	How much does this child (name) weigh NOW?	_______ kilograms ______ grams<br><br>I don't know
22.	How was this child (name) delivered?	1) Vaginal<br>2) Planned Cesarean<br>3) Acute/Induced Cesarean
23.	What is the birth date of this child (name)?	(dd/mm/yyyy) _____/_____/__________

	Question	Answer
24.	How many children who live in your house (include step children and other's children) including this child (name) are below 18 years old?	_____________
25.	Besides yourself, and not counting your children under 18, how many other people live in your house. *Include siblings, cousins, other relatives, and non-relatives in the count to get an estimate of household size.* __________	_____________
**PART IIA : CHILD FEEDING KNOWLEDGE, ATTITUDES AND PRACTICE AMONG MOTHERS**		
	Question	Answer
26.	Have you ever breastfed this child (name)?<br>*("Ever" means if mother has breast fed at all to this baby)*	1) Yes<br>2) No **(Skip to Q31)**
27.	Are you breastfeeding this child (name) now?	1) Yes<br>2) No (if no, what was the age of the baby when you stopped------------months
28.	Are you exclusively breastfeeding (no water or liquid) this child now?	1) Yes<br>2) No ((if no, what was the age of the baby when you stopped-----------months
	Next two questions apply to timing of discontinuation or termination of exclusive breastfeeding	
29.	How old was this child (name) when you stopped **exclusively breastfeeding (only mother's milk)** this child? ***For mothers who but have STOPPED Exclusively breastfeeding this baby at the time of interview but are giving mother's milk along with other food (i.e. formula, solid food etc)***	___________ in months ________ days
30.	How old was this child (name) when you stopped **giving any breast milk** to this child? ***For mothers who stopped giving any milk***	___________ in months ________ days
31.	What are you feeding your child now?<br>***For mothers who are NOT breastfeeding now AND NEVER breastfed this baby (answered "No" to BOTH Q26 AND Q27)***	1) Formula only<br>2) Formula and solid & liquid food<br>3) Solid and liquid food – No formula
32.	***For mothers who are partially breastfeeding their child now at the time of interview. For mothers who answered Yes to Ever breastfed (Q26) and Yes to currently breastfeeding (Q27)***<br>What are you feeding your child now?	1) Breast milk and formula<br>2) Formula only<br>3) Breast milk, formula and other solid & liquid food<br>4) Formula and solid & liquid food<br>5) Solid and liquid food ONLY– No formula
33.	How soon did you start breastfeeding after delivery of this child (name)? ***For mothers who answered Yes to Ever breastfed (Q26) and Yes to currently breastfeeding (Q27)***	1) In less than an hour after delivery<br>2) About an hour or so after delivery<br>3) 1-2 hours after delivery<br>4) 2.5 – 5 hours after delivery<br>5) 5-10 hours after delivery<br>6) >10 hours after delivery

	Question	Answer
		7) Started breastfeeding at home after discharge from hospital
34	If you have more than one child, did you breastfeed your older child(children) (Applies to all respondents)	1. This is my first child<br>2. No, I never breastfed my any other child(ren)<br>3. Yes, breastfed some of my children<br>4. Yes, I breastfed all my children
35	Did you ever think of breastfeeding this child (name) when you were pregnant with this child (name)?	1) No<br>2) Yes
36	**If "Yes" to Q35,** which month of your pregnancy did you start thinking that you may breastfeed this child (name)?	1) First 3 months<br>2) 4-6 months<br>3) 7 months or later<br>4) Decided to breastfeed soon after my child was born<br>5) Don't remember
37	Did you get support and encouragement from family members and relatives on breastfeeding	1) No<br>2) Yes
38	**If "Yes" to Q37,** who are the people in your family who support and encourage you to breastfeeding this child (**Check all that apply)**	1) Mother-in Law<br>2) Mother<br>3) Husband<br>4) Other relatives (specify)________________<br>5) Other non-relatives (specify) ______________
39	Did your child (name) stay with you in the same room during your hospital stay (Rooming-in)	1) No<br>2) Yes
40	To the best of your knowledge, for how long should a mother breastfeed her baby?	1) Don't have to breastfeed<br>2) Less than 6 months<br>3) 6 to 12 months<br>4) 13 to 18 months<br>5) 18 to 24 months<br>6) 24 months<br>7) More than 24 months<br>8) Don't know
41	To the best of your knowledge, at what age (of the child) should a mother start giving solid food?	1) Less than 6 months<br>2) 6 months<br>3) More than 6 months<br>4) I don't know
42	Where do you usually get information about breastfeeding? (**Check all that apply**)	1) Hospital<br>2) Health Center or Clinic<br>3) Family members<br>4) Other Relatives<br>5) Friends and Non-Relatives<br>6) Television/Radio

	Question	Answer
		7) Social Media<br>8) Others (specify)
43	While you were pregnant with this child, did someone discuss and/or advise to you about breastfeeding?	1) No<br>2) Yes
44	If Yes to Q43, then please name the person(s) who discussed and/or advised to you on positive or negative things about breastfeeding during your pregnancy with this child. **Check all that apply.**	Discussed/Advised<br>Positive Negative<br>1) My Obstetrician/Nurse/Health Professional at the hospital/Clinic ____ ____<br>2) A Lactation Specialist at the hospital/Clinic ____ ____<br>3) My Mother ____ ____<br>4) My in-Laws (includes mother in-law) ____ ____<br>5) My Husband ____ ____<br>6) Other Relatives ____ ____<br>7) Friends and other non-relatives ____ ____<br>8) Others (Specify)________________ ____ ____
45	After you delivered this child, did someone discuss and/or advise to you about breastfeeding?	1) No<br>2) Yes
46	If Yes to Q45, then please name the person(s) who discussed and/or advised to you on positive or negative things about breastfeeding after you delivered this child. **Check all that apply.**	Discussed/Advised<br>Positive Negative<br>1) My Obstetrician/Nurse/Health Professional at the hospital/Clinic ____ ____<br>2) A Lactation Specialist at the hospital/Clinic ____ ____<br>3) My Mother ____ ____<br>4) My in-Laws (includes mother in-law) ____ ____<br>5) My Husband ____ ____<br>6) Other Relatives ____ ____<br>7) Friends and other non-relatives ____ ____<br>8) Others (Specify)________________ ____ ____
	**PART IIB. COMPLEMENTARY FEEDING (ASK ONLY TO MOTHERS WHO HAVE STOPPED EXCLUSIVELY BREASTFEEDING THIS CHILD)**	
47	At what age did you start giving your child water?	1) Less than 1 month<br>2) 1 month<br>3) 2 months<br>4) 3 months<br>5) 4 months<br>6) 5 months<br>7) 6 months<br>8) More than 6 months
48	What was the age of your child when you started complementary foods (CF) such as cereal, meat, fish,	1) Less than 6 months<br>2) 6 months

	Question	Answer
	yoghurt, egg, cheese, fruits and vegetables etc?	3) More than 6 months<br>4) Formula feed only<br>5) Have not started yet<br>6) I don't know
49	What type of food(s) have you been giving your baby as complementary food (CF)? **Check all that apply**	1. animal proteins (for example: meat, fish, chicken, liver, egg..etc.)<br>2. milk proteins (for example: yoghurt, cheese, milk ….etc.)<br>3. plant proteins (pulses, lentils and legumes)<br>4. cereals<br>5. fats and oil<br>6. vegetables (for example: red, dark green or orange) or<br>7. fruits (red or orange fruits, dates)<br>8. cakes, chocolates and sweets<br>9. chips , crisps,and other salty food<br>10. other (specify)____
50	How many times do you currently give your child Complementary Food in 24 hours (one day)?	________ times
51	What do you typically include in a main meal for this child? **(Open ended, Will code them during data input)**	_________________
52	How much CF do you give to your child at each meal? (show a 250 ml bowel or cup)	1\2 of a 250 ml cup<br>2/3 of a 250 ml cup<br>3/4 of a 250 ml cup<br>A full 250 ml cup<br>Others (specify)
53	What type of food do you give to your child as a snack? (**Open ended, Will code them during data input)**	_________________ _________________ _________________
54	How many times do you give your child snacks in a day?	________ times
55	What type of drinks /beverages do you give to your child as a snack? **(Open ended, Will code them during data input)**	_________________
56	How many times do you give your child beverages/drinks in a day? Do not include breastfeeding or fomualr	________ times
